# Supplementary material for: Deep Learning in Alzheimer's Disease: Diagnostic Classification and Prognostic Prediction Using Neuroimaging Data
Source: Front Aging Neurosci. 2019 Aug 20;11:220. doi: 10.3389/fnagi.2019.00220 (PMC6710444; doi:10.3389/fnagi.2019.00220)
Supplement: Supplementary file 1 [file Data_Sheet_1.pdf]

Supplementary Material

Deep Learning in Alzheimer's Disease: Diagnostic Classification and Prognostic Prediction using Neuroimaging Data

Taeho Jo[1-3*], Kwangsik Nho[1-3], Andrew J. Saykin[1-3]

[1]Center for Neuroimaging, Department of Radiology and Imaging Sciences, Indiana University School of Medicine, Indianapolis, IN, USA[2]Indiana Alzheimer Disease Center, Indiana University School of Medicine, Indianapolis, IN, USA
[3]Indiana University Network Science Institute, Bloomington, IN, USA

*** Correspondence:** Corresponding Author tjo@iu.edu

Supplement 1. Weights calculation in the backpropagation

After the initial error value is calculated from the given random weight by the least squares method, the weights are updated until the differential value becomes 0. The differential value 0 means there is no change in weight when the gradient is subtracted from the previous weight. In Fig. 1, the w_{31} is updated by following formula:

$$\boldsymbol{w_{31}(t+1) = w_{31}t - \frac{\partial ErrorY_{out}}{\partial w_{31}}}$$

$$\boldsymbol{ErrorY_{out} = \frac{1}{2}(y_{t1} - y_{o1})^2 + \frac{1}{2}(y_{t2} - y_{o2})^2}$$

The *Error*Y_{out} is the sum of error y_{o1} and error y_{o2}. y_{t1}, y_{t2} are constants that are known through the given data. The partial derivative of *Error*Y_{out} with respect to w_{31} can be calculated by the chain rule as follows.

$$\boldsymbol{\frac{\partial ErrorY_{out}}{\partial w_{31}} = \underbrace{\frac{\partial ErrorY_{out}}{\partial y_{o1}}}_{\text{(i)}} \cdot \underbrace{\frac{\partial y_{o1}}{\partial net3}}_{\text{(ii)}} \cdot \underbrace{\frac{\partial net3}{\partial w_{31}}}_{\text{(iii)}}}$$

Here, (i) becomes y_{o1}-y_{t1} which is the partial derivative of $\frac{1}{2}(y_{t1} - y_{o1})^2$ with respect to y_{o1}. When activation function σ(x) is $\frac{1}{1+e^{-x}}$, $\frac{d\sigma(x)}{dx}$= σ(x)·(1-σ(x)) which makes (ii) y_{o1}·(1-y_{o1}). Since Net_3 is $w_{31}y_{h1}$ + $w_{41}y_{h2}$ + bias, the partial derivative of Net_3 with respect to w_{31}, which (iii), is y_{h1}.

$$\boldsymbol{w_{31}(t+1) = w_{31}t - (y_{o1} - y_{t1})y_{o1}(1 - y_{o1})y_{h1}}$$

To update W_{11} in hidden layer, it is also started from *Error*Y_{out}, since Y_h is located in the hidden layer and is not exposed.

$$w_{11}(t+1) = w_{11}t - \frac{\partial ErrorY_{out}}{\partial w_{11}}$$

$$\frac{\partial ErrorY_{out}}{\partial w_{11}} = \frac{\partial ErrorY_{out}}{\partial y_{h1}} \cdot \frac{\partial y_{h1}}{\partial net_1} \cdot \frac{\partial net_1}{\partial w_{11}}$$

(i)

Here, the calculation of (i) is a bit different from previous. Since *Error*Y_{out} includes *Error*y_{o1} and *Error*y_{o2}, it is calculated as follows.

$$\frac{\partial ErrorY_{out}}{\partial h_1} = \frac{\partial (Errory_{o1} + Errory_{o2})}{\partial y_{h1}} = \frac{\partial Errory_{o1}}{\partial y_{h1}} + \frac{\partial Errory_{o2}}{\partial y_{h1}}$$

(a) (b)

(a), (b) is calculated as follows by the chain rule.

(a) $$\frac{\partial Errory_{o1}}{\partial y_{h1}} = \frac{\partial Errory_{o1}}{\partial net_3} \cdot \frac{\partial net_3}{\partial y_{h1}} = (y_{o1} - y_{t1}) y_{o1} (1 - y_{o1}) y_{o1}$$

(b) $$\frac{\partial Errory_{o2}}{\partial y_{h1}} = \frac{\partial Errory_{o2}}{\partial net_4} \cdot \frac{\partial net_4}{\partial y_{h1}} = (y_{o2} - y_{t2}) y_{o2} (1 - y_{o2}) y_{o2}$$

Now, (i), (ii), and (iii) are summarized as follows.

$$\frac{\partial ErrorY_{out}}{\partial w_{11}} = \frac{\partial ErrorY_{out}}{\partial y_{h1}} \cdot \frac{\partial y_{h1}}{\partial net_1 y} \cdot \frac{\partial net_1}{\partial w_{11}}$$

$$= (\delta y_{o1} y_{o1} - \delta y_{o2} y_{o2}) y_{h1} (1 - y_{h1}) x_1$$

Supplement 2. Advanced gradient descent methods

Nesterov Momentum is the method of adding the value of $\gamma v_{(t-1)}$ to the Momentum SGD to find the gradient. This allows to reduce unnecessary movements by advance movement in the direction to move.

$$w_{(t+1)} = w_t + \gamma v_{(t-1)} - \eta \frac{\partial Error}{\partial (w + \gamma v_{(t-1)})}$$

Adagrad(Adaptive Gradient) is an optimization method that adjusts the learning rate according to the number of update of variables.

$$\boldsymbol{G_t = G_{(t-1)} + \left(\frac{\partial Error}{\partial w_t}\right)^2}$$

$$\boldsymbol{w_{(t+1)} = w_t - \eta \frac{1}{\sqrt{G_t + \epsilon}} \frac{\partial Error}{\partial w_t}}$$

Here, RMSprop is the method of adjusting the ratio between the previous value and the modified value.

$$\boldsymbol{G_t = \gamma G_{(t-1)} + (1-\gamma)\left(\frac{\partial Error}{\partial w_t}\right)^2}$$

$$\boldsymbol{w_{(t+1)} = w_t - \eta \frac{1}{\sqrt{G_t + \epsilon}} \frac{\partial Error}{\partial w_t}}$$

Adam, the most popular optimization method for deep learning today, takes advantage of momentum SGD and RMSprop. Adam is expressed as follows. Where G_t is the sum of the square of the modified gradient. ε is a very small constant that prevents it from being divided by zero.

$$\boldsymbol{V_t = \gamma G_{(t-1)} + (1-\gamma_1) \frac{\partial Error}{\partial w_t}}$$

$$\boldsymbol{G_t = \gamma G_{(t-1)} + (1-\gamma_2)\left(\frac{\partial Error}{\partial w_t}\right)^2}$$

$$\boldsymbol{\widehat{V}_t = \frac{V_t}{1-\gamma_1^t} \quad \widehat{G}_t = \frac{G_t}{1-\gamma_2^t}}$$

$$\boldsymbol{w_{(t+1)} = w_t - \eta \frac{\widehat{G}_t}{\sqrt{\widehat{V}_t + \epsilon}}}$$

Supplement 3.

Table S1. All the results of the 16 studies to systematically be reviewed

Author (year)	Modality	Data processing, training	Classifier	AD	cMCI	ncMCI	NC	Total	Acc. AD/NC	STD	Acc. MCI conversion	STD
**Suk et al. (2015)**	MRI,PET,CSF	SAE + sparse learning	SVM	51	43	56	52	202	**98.8**	0.4	**83.3**	2.1
**Choi and Jin (2018)**	PET	3D CNN	softmax	139	79	92	182	492	**96**		**84.2**	
**Suk and Shen (2013)**	MRI,PET,CSF	SAE	SVM	51	43	56	52	202	**95.9**	1.1	**75.8**	2
**Suk et al. (2014)**	MRI,PET	DBM	SVM	93	76	128	101	398	**95.35**	5.23	**75.92**	15.37
**Li et al. (2014)**	MRI, PET	3D CNN	Logistic regression	198	167	236	229	830	**92.87**	2.07	**72.44**	2.41
**Suk et al. (2015)**	MRI	SAE + sparse learning	SVM	51	43	56	52	202	**92.4**	1.5	**69.3**	2
**Suk et al. (2014)**	MRI	DBM	SVM	93	76	128	101	398	**92.38**	5.32	**72.42**	13.09
**Suk et al. (2014)**	PET	DBM	SVM	93	76	128	101	398	**92.2**	6.7	**70.25**	13.23
**Li et al. (2014)**	MRI	3D CNN	Logistic regression	198	167	236	229	830	**91.92**	1.88	**71.68**	2.53
**Aderghal et al. (2017)**	MRI	2D CNN	softmax	188	399		228	815	**91.41**			
**Li et al. (2015)**	MRI,PET,CSF	RBM + Drop out	SVM	51	43	56	52	202	**91.4**	1.8	**57.4**	3.6
**Liu et al. (2015)**	MRI,PET	SAE with zero-masking	softmax	77	67	102	85	331	**91.4**	5.56		
**Liu et al. (2018a)**	PET	RNN	softmax	93	146		100	339	**91.2**			
**Vu et al. (2017)**	MRI, PET	SAE + 3D CNN	softmax	145			172	317	**91.14**			
**Liu et al. (2018b)**	MRI	Landmark detection + 3D CNN	softmax	159	38	239	200	636	**91.09**		**76.9**	
**Cheng and Liu (2017)**	MRI,PET	3D CNN + 2D CNN	softmax	93			100	193	**89.64**			
**Suk et al. (2015)**	PET	SAE + sparse learning	SVM	51	43	56	52	202	**88.7**	2.7	**68.9**	3.8
**Li et al. (2014)**	PET	3D CNN	Logistic regression	198	167	236	229	830	**87.62**	2.36	**70.29**	2.45
**Liu et al. (2014)**	MRI,PET	SAE+NN	softmax	65	67	102	77	311	**87.76**			
**Cheng et al. (2017)**	MRI	3D CNN	softmax	199			229	428	**87.15**			
**Cheng and Liu (2017)**	PET	3D CNN + 2D CNN	softmax	93			100	193	**87.13**			
**Cheng and Liu (2017)**	MRI	3D CNN + 2D CNN	softmax	93			100	193	**85.47**			
**Lu et al. (2018)**	MRI,PET	DNN + NN	softmax	238	217	409	360	1224	**84.6**	1.5	**82.93**	7.25
**Lu et al. (2018)**	PET	DNN + NN	softmax	238	217	409	360	1224	**84.5**	1.4	**81.53**	7.42
**Lu et al. (2018)**	MRI	DNN + NN	softmax	238	217	409	360	1224	**81.9**	1.2	**75.44**	7.74
**Korolev et al. (2017)**	MRI	3D CNN	softmax	50			61	111	**80**	7		
**Suk et al. (2015)**	CSF	SAE + sparse learning	SVM	51	43	56	52	202	**79.7**	1.4	**57.7**	3

All data on this table were from ADNI.
https://github.com/rasmusbergpalm/DeepLearnToolbox (Suk et al. (2015), Suk and Shen (2013))
https://github.com/neuro-ml/resnet_cnn_mri_adni (Korolev et al. (2017))

Reference

Aderghal, K., Benois-Pineau, J., Afdel, K., and Catheline, G. (2017). "FuseMe: Classification of sMRI images by fusion of Deep CNNs in 2D+E projections", in: *Proceedings of the 15th International Workshop on Content-Based Multimedia Indexing.*

Cheng, D., Liu, M., Fu, J., and Wang, Y. (2017). "Classification of MR brain images by combination of multi-CNNs for AD diagnosis", in: *Ninth International Conference on Digital Image Processing (ICDIP 2017)*: SPIE), 5.

Cheng, D., and Liu, M. (2017). "CNNs based multi-modality classification for AD diagnosis", in: *2017 10th International Congress on Image and Signal Processing, BioMedical Engineering and Informatics (CISP-BMEI)*), 1-5.

Choi, H., and Jin, K.H. (2018). Predicting cognitive decline with deep learning of brain metabolism and amyloid imaging. *Behavioural Brain Research* 344**,** 103-109.

Korolev, S., Safiullin, A., Belyaev, M., and Dodonova, Y. (2017). "Residual and plain convolutional neural networks for 3D brain MRI classification", in: *2017 IEEE 14th International Symposium on Biomedical Imaging (ISBI 2017)*), 835-838.

Li, R., Zhang, W., Suk, H.-I., Wang, L., Li, J., Shen, D., and Ji, S. (2014). Deep learning based imaging data completion for improved brain disease diagnosis. *Medical image computing and computer-assisted intervention : MICCAI ... International Conference on Medical Image Computing and Computer-Assisted Intervention* 17**,** 305-312.

Li, F., Tran, L., Thung, K.-H., Ji, S., Shen, D., and Li, J. (2015). A Robust Deep Model for Improved Classification of AD/MCI Patients. *IEEE journal of biomedical and health informatics* 19**,** 1610-1616.

Liu, M., Cheng, D., Yan, W., and , A.S.D.N.I. (2018a). Classification of Alzheimer’s Disease by Combination of Convolutional and Recurrent Neural Networks Using FDG-PET Images. *Frontiers in Neuroinformatics* 12.

Liu, S., Liu, S., Cai, W., Pujol, S., Kikinis, R., and Feng, D. (2014). "Early diagnosis of Alzheimer's disease with deep learning", in: *2014 IEEE 11th International Symposium on Biomedical Imaging (ISBI)*), 1015-1018.

Liu, S., Liu, S., Cai, W., Che, H., Pujol, S., Kikinis, R., Feng, D., Fulham, M.J., and Adni (2015). Multimodal Neuroimaging Feature Learning for Multiclass Diagnosis of Alzheimer’s Disease. *IEEE Transactions on Biomedical Engineering* 62**,** 1132-1140.

Liu, M., Zhang, J., Adeli, E., and Shen, D. (2018b). Landmark-based deep multi-instance learning for brain disease diagnosis. *Medical Image Analysis* 43**,** 157-168.

Lu, D., Popuri, K., Ding, G.W., Balachandar, R., and Beg, M.F. (2018). Multimodal and Multiscale Deep Neural Networks for the Early Diagnosis of Alzheimer’s Disease using structural MR and FDG-PET images. *Scientific Reports* 8**,** 5697.

Suk, H.-I., and Shen, D. (2013). Deep Learning-Based Feature Representation for AD/MCI Classification. *Medical image computing and computer-assisted intervention : MICCAI ...*

International Conference on Medical Image Computing and Computer-Assisted Intervention 16**,** 583-590.

Suk, H.-I., Lee, S.-W., Shen, D., and The Alzheimers Disease Neuroimaging, I. (2014). Hierarchical Feature Representation and Multimodal Fusion with Deep Learning for AD/MCI Diagnosis. *NeuroImage* 101**,** 569-582.

Suk, H.-I., Lee, S.-W., Shen, D., and The Alzheimer's Disease Neuroimaging, I. (2015). Latent feature representation with stacked auto-encoder for AD/MCI diagnosis. *Brain structure & function* 220**,** 841-859.

Vu, T.D., Yang, H.-J., Nguyen, V.Q., Oh, A.R., and Kim, M.-S. (2017). "Multimodal learning using convolution neural network and Sparse Autoencoder", in: *2017 IEEE International Conference on Big Data and Smart Computing (BigComp)*), 309-312.
